# Reliability of a Low-Cost Inertial Measurement Unit (IMU) to Measure Punch and Kick Velocity

**DOI:** 10.3390/s25020307

**Published:** 2025-01-07

**Authors:** Lukas Pezenka, Klaus Wirth

**Affiliations:** Institute of Sport Science, University of Applied Sciences Wiener Neustadt, 2700 Wiener Neustadt, Austria; klaus.wirth@fhwn.ac.at

**Keywords:** combat sports, kinematics, reliability, accelerometry

## Abstract

Striking velocity is a key performance indicator in striking-based combat sports, such as boxing, Karate, and Taekwondo. This study aims to develop a low-cost, accelerometer-based system to measure kick and punch velocities in combat athletes. Utilizing a low-cost mobile phone in conjunction with the PhyPhox app, acceleration data was collected and analyzed using a custom algorithm. This involved strike segmentation and numerical integration to determine velocity. The system demonstrated moderate reliability (intraclass correlation coefficient (ICC) 3,1 = 0.746 to 0.786, standard error of measurement (SEM) = 0.488 to 0.921 m/s), comparable to commercially available systems. Biological and technical variations, as well as test standardization issues, were acknowledged as factors influencing reliability. Despite a relatively low sampling frequency, the hardware and software showed potential for reliable measurement. The study highlights the importance of considering within-subject variability, hardware limitations, and the impact of noise in software algorithms. Average strike velocities exhibited higher reliability than peak velocities, making them a practical choice for performance tracking, although they may underestimate true peak performance. Future research should validate the system against gold-standard methods and determine the optimal sampling frequency to enhance measurement accuracy.

## 1. Introduction

Combat sports (CS) have a rich history. Records from ancient Greece show that CS were practiced as early as 649 B.C. [1,2,3]. Today, CS are predominant events at the Olympic games, with approximately 16% of total medals being awarded in a CS discipline [4].

In the class of striking-based CS, such as boxing, Karate, and Taekwondo, striking velocity plays an important role and can be considered a key performance indicator (KPI) [5]. In competitions that can be won by knockout, striking velocity is of particular importance, as it has been shown to influence the impact force applied to an opponent [6]. Furthermore, as pointed out by Corcoran et al. [7], quicker strikes have a higher chance of bypassing an opponent’s defenses.

Straight punches and roundhouse kicks are amongst the most common offensive techniques in kickboxing [8]. Different methods of impact force [9] and velocity measurements for punches and kick have been proposed in the literature.

Marker-based motion tracking systems are considered the gold standard for tracking human movement [10,11]. In a recent review, Corcoran et al. [7] reported that camera-based motion capture systems were most frequently used in the literature for kicking velocity measurement, followed by ground sensors and racket systems and dual-beam laser systems. However, while high-quality camera-based systems offer precise measurements, they are often expensive and lack portability, making them less accessible to many practitioners.

The advent of low-cost electronics, small battery sizes, and wireless data logging, which are the characteristics of inertial measurement units (IMUs), has made non-intrusive, ubiquitous sports monitoring equipment widely available [12]. In particular, wearable technology for physical activity recognition has seen a surge in research interest in recent years [13].

A recent review on the validity and reliability of smartphone accelerometers in the context of gait analysis [14] suggested that smartphone-based sensors can offer a reliable and cost-effective solution for motion analysis in ecologically valid environments. These sensors have been validated against gold-standard technologies, such as motion capture systems, and have demonstrated acceptable accuracy and reliability.

In combat sports research, a wide array of research applications has recently emerged. Worsey et al. [15] conducted a systematic review on the use of IMUs for performance analysis in combat sports and concluded that strike quality was the feature of most interest in the related literature. IMUs can be used in two ways in the context of strike analysis: either attached to the target, where acceleration data can be used to gauge impact, or as wearable devices, which register the acceleration of the studied limb directly.

Kimm and Thiel [16] implemented an accelerometer-based measurement system to investigate the influence of experience and reach on the velocity of jab and and cross punches. Similarly, Tiwari et al. [17] used a triaxial accelerometer attached to a boxing glove in order to analyze kinematic parameters, such as velocity, acceleration, and angular rotation in straight and hook punches. Jovanovski and Stappenbelt [18] used a triaxial accelerometer in conjunction with a piezo-resistive sensor in a boxing glove to measure velocity prior to impact and punching force. The authors pointed out that the main drawback of the proposed measurement system comes from the piezo-resistive sensor, whose size and placement compromised validity and did not allow the athlete to demonstrate all punching techniques, due to insufficient coverage.

Lambert et al. [19] compared the reliability of a commercially available accelerometer and a commercially available linear position transducer for measuring punch speed and acceleration. The authors reported excellent intra-device reliability for maximum punch trials for both devices. Both the linear position transducer (r = 0.918 to 0.949; ICC = 0.977, 95% confidence interval (CI) [0.960, 0.987], *p* = 0.001) and the accelerometer (r = 0.866 to 0.924; ICC = 0.959, 95% CI [0.929, 0.977], *p* = 0.001) showed strong positive associations between repeated measures for maximum velocity. The limits of agreement (LOAs) between the linear position transducer and the accelerometer were −1.36 m/s to 1.62 m/s for maximum velocity and −16.27 m·$s^{-2}$ to 8.73 m·$s^{-2}$ for maximum acceleration. The mean bias between devices was found to be 0.13 m/s for maximum velocity and −3.77 ${\mathrm{ms}}^{-2}$ for maximum acceleration. When interpreting these results, it needs to be kept in mind that no gold standard was referenced in this investigation.

Similarly, Harris et al. [5] analyzed the reliability of a commercially available accelerometer and a commercially available linear position transducer for measuring punch speed for the rear straight punch. The linear position transducer demonstrated good reliability for mean peak (ICC3,1 = 0.871, 95% CI [0.689, 0.95]) and maximum peak (ICC3,1 = 0.853, 95% CI [0.65, 0.942]) velocity. Conversely, the accelerometer presented weak reliability for mean peak (ICC3,1 = 0.309, 95% CI [−0.17, 0.67]) and maximum peak (ICC3,1 = 0.227, 95% CI [−0.173, 0.637]) velocity.

López-Laval et al. [20] validated a commercially available punch tracker by comparing the measured punch velocity to the results of manual analysis with the Kinovea^®^ biomechanical video analysis software. Intra-device measurement reliability of the punch tracker was good for the left arm (ICC = 0.78, 95% CI [0.69, 0.86], *p* < 0.001) and moderate for the right arm (ICC = 0.63, 95% CI [0.51, 0.75], *p* < 0.001). Furthermore, the correlation between the results obtained from the tracker and those from the Kinovea^®^ software was strong for both the right arm (r = 0.9, 95% CI [0.88, 0.92], *p* < 0.001) and left arm (r = 0.92, 95% CI [0.9, 0.94], *p* < 0.001). From these results, the authors concluded that the punch tracker can be used to assess the maximal velocity of straight punches.

In summary, while accelerometers have shown promise in previous research, their reliability may be less consistent than initially assumed. The correlation coefficients reported across studies exhibit considerable variation, and the Bland–Altman plots indicate potential for significant deviations between measurements. These findings suggest that, while accelerometers are useful tools, their measurements should be interpreted with caution, especially in applications requiring high precision. Hence, the aim of this study was to assess the reliability of an IMU-based velocity measurement system that is based on common and readily available hardware (i.e., a low-cost smartphone) and free, open access software tools, in conjunction with a custom algorithm implemented in the Python programming language.

## 2. Materials and Methods

### 2.1. Subjects

Forty-three (27 men and 16 women) healthy, trained CS athletes from Vienna, Austria with at least one year of experience in either kickboxing or Mixed Martial Arts (MMA) volunteered to participate in this study. Five subjects dropped out of the study before completing all sessions due to injury or time commitment problems. Hence, 38 (26 men and 12 women) subjects completed the study. The anthropometric data of the athletes who completed the study are shown in Table 1. Before the study, all subjects were informed of the risks and potential benefits of the investigation. They gave informed written consent to participate in the study. The study was conducted in accordance with the Declaration of Helsinki and was approved by the Institutional Review Board of University of Applied Science Wiener Neustadt (RB20221005011, 5 October 2022).

### 2.2. Procedures

Subjects attended a strength room for testing on two occasions that were separated by two weeks. Familiarization and the first testing occurred in the first week. On both testing days, participants were instructed to perform a self-directed warm-up based on their own training routines, reflecting their familiarity with effective warm-up techniques. No standardized warm-up protocol was imposed, as all participants had a minimum of one year of training experience and were encouraged to prepare according to their personal preferences to ensure readiness for testing.

Although there were methodological differences (such as sensor placement) in measuring upper and lower limb speed, the conceptual approach was identical for both.

Absolute values (i.e., the accelerometer norm) were utilized for velocity estimation. Although sensor fusion and pose estimation using accelerometer–gyroscope combinations [11,21,22] are a more sophisticated approach for analyzing human movement, the use of absolute acceleration has been proposed as an alternative in the literature for high-speed movements, such as punches and kicks [16,23]. This method, as discussed by Kimm and Thiel [16] and Socci et al. [23], involves calculating the magnitude of the acceleration vector and identifying the point of impact, which is then used to segment the data.

Acceleration during striking was measured via the built-in accelerometer of a smartphone (P8 Lite, Huawei Technologies Co., Ltd., Shenzhen, China) with an integrated IMU (LIS3DH, STMicroelectronics, Plan-les-Ouates, Switzerland).

To our knowledge, the LIS3DH sensor has not been validated against a gold-standard method, such as motion capture, for tracking high-speed movements. However, accelerometers from the same manufacturer as the LIS3DH sensor have been validated in previous studies. A recent study [24] demonstrated that smartphones with accelerometers from the same manufacturer as the LIS3DH were valid and reliable for estimating accelerations in dynamic activities, such as lower limb motion during gait trials. Accelerations were calculated using the Vicon MX motion capture system (Vicon Motion Systems, Oxford, UK), a widely accepted gold standard for assessing human movement [10,11]. The study found no significant differences between smartphones and the motion capture system (p>0.05), with ICC values ranging from poor to good (ICC = −0.348 to 0.796), indicating variability in reliability across devices. The authors concluded that smartphone accelerometers are valid for estimating accelerations in dynamic activities.

Another study [25] validated a Huawei G620S smartphone (Huawei Technologies Co., Ltd., Shenzhen, China) with an LIS3DH sensor against a Speed4Lifts (Speed4Lifts, Madrid, Spain) linear transducer for measuring concentric barbell velocity. The authors found good agreement between the two devices (ICC = 0.634; CI [0.308, 0.794]; Cronbach’s alpha = 0.698), with no significant differences for lifts performed at 70% 1RM (p>0.05). However, for heavier lifts (>70% 1RM), significant differences were observed, with lower movement speeds affecting the measurements. The authors concluded that the smartphone accelerometer is reliable for velocities around 0.52±0.11 m/s but less reliable at lower velocities.

Similarly, research [26] on a comparable smartphone from the same manufacturer showed an average error rate of 0.16% when detecting repetitions in resistance training, further supporting the validity of accelerometers from this manufacturer for dynamic movement analysis.

The PhyPhox app (RWTH Aachen University, Aachen, Germany) [27], which was shown to lead to valid and reliable data [28], was used to record the acceleration data at a sampling rate of 50 Hz. This sampling frequency was identified by Worsey et al. [15] as the lower threshold for analyzing movement speed with inertial sensors. Strictly speaking, this sampling frequency does not satisfy the Nyquist–Shannon sampling theorem, which will be discussed in Section 4 in more detail.

**Figure 1:**
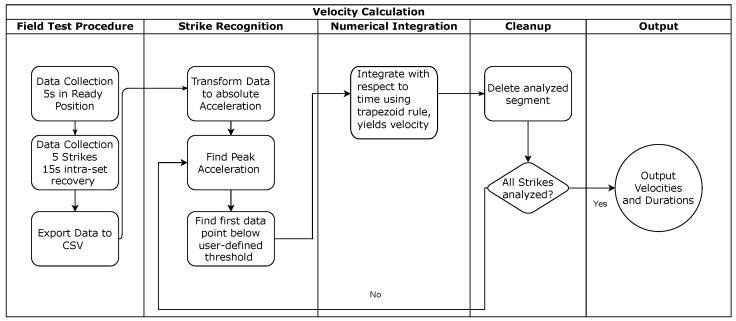
Methodological approach to velocity calculation. Data are collected during the field test and then segmented and evaluated in a post-processing step. CSV: comma separated file, s: second.

Offline analysis was performed using a custom algorithm written in the Python programming language [29]. The analysis consisted of strike segmentation and numerical integration in order to obtain the striking velocity. Figure 1 illustrates the process of segmenting a strike from the acceleration data and subsequently calculating the velocity.

Acceleration data was collected on the smartphone and wirelessly transmitted to the controlling laptop computer. The resulting comma separated file (CSV) file was then post-processed on a personal computer and, after pseudonymization, the computation results were copied into a spreadsheet for statistical analysis at a later point in time.

Before the test, the subjects took on a ready position, with the hands in the proper guard position, at the right distance to the bag. In this position, five seconds of acceleration data (noise) were recorded. These data were later used to identify a threshold for strike recognition.

After five seconds of data collection, the subjects executed a total of 5 strikes with the lead limb. Participants were instructed to perform each strike at maximum speed while adhering to specific restrictions: no footwork or trunk rotation was allowed. All five strikes (jabs or kicks) were directed consistently at the same target area on the punching bag to standardize the impact location across repetitions. This approach ensured that each strike was as similar as possible in execution, minimizing variability in technique and target position.

Resting times were at least 10 s between strikes and at least 15 min between punches and kicks. After each strike, the subjects returned to the initial position and waited for the next command. As described by López-Laval et al. [20], if abnormalities were detected in the execution or if errors happened in the accelerometer reading, repetitions were considered invalid.

Five repetitions were chosen to balance the need for reliable data with the goal of minimizing fatigue, which is particularly relevant in high-power, fast movements like striking. Existing research on explosive actions, such as countermovement jumps (CMJs), has shown that performance can decline after only a few repetitions. For instance, a recent study demonstrated that peak power output decreases as early as the second jump [30]. Although a circular kick involves less mass than a CMJ, likely leading to less pronounced fatigue effects, performance degradation with increased repetition was still anticipated. Therefore, five repetitions were selected to limit fatigue-related impacts on performance while providing an adequate sample size for analysis.

Additionally, the inherent variability in performing complex, technically demanding movements like strikes was considered. Highly technical movements often exhibit greater variation, and the first repetition is not necessarily the best representation of an athlete’s peak performance. Variability in performance is a concern when measuring highly technical skills such as strikes, as shown by the within-day coefficient of variation (CV) for strike performance, which ranged from 0.67% to 17.81% (see Section 3). This level of variation suggests that some participants found the task challenging and had not yet achieved a stable, repeatable technique. Since only one year of training experience was required for inclusion in the study, technical inconsistency was anticipated. Allowing each participant five repetitions provided multiple opportunities to capture their best strike, which was particularly important for participants who might require more attempts to reach their peak execution.

The collected acceleration data was stored in a CSV file and subsequently passed on to the segmentation algorithm. There, during a preparatory step, the magnitude of acceleration across all dimensions $A=\sqrt{A_{X}^{2}+A_{Y}^{2}+A_{Z}^{2}}$ was calculated for each discrete time point. Then, the peak (i.e., maximum acceleration) $A_{peak}$ was identified. As explained by Socci et al. [23], $A_{peak}$ represents the instant of impact of the strike on the bag.

**Figure 2:**
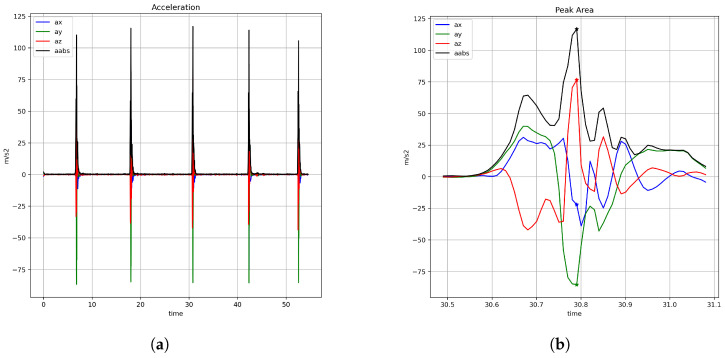

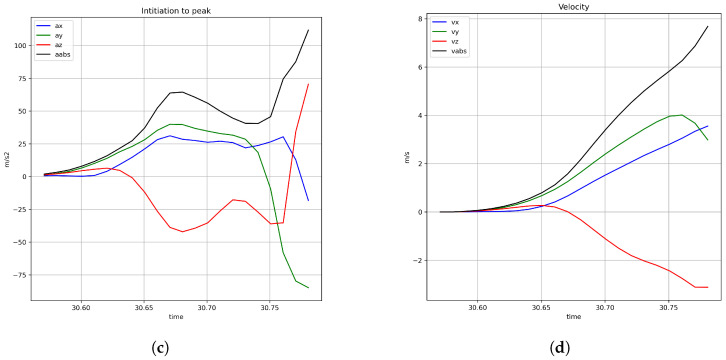
Strike segmentation and velocity calculation. m: meter, s: second, ms: millisecond, a: acceleration, xyz: respective axis of motion, aabs: absolute acceleration ($\sqrt{a_{x}^{2}+a_{y}^{2}+a_{z}^{2}}$), v: velocity, vabs: absolute velocity ($\sqrt{v_{x}^{2}+v_{y}^{2}+v_{z}^{2}}$). (**a**) Acceleration profiles of five punches; (**b**) segmented punch (600 ms around peak acceleration); (**c**) initiation to contact; (**d**) velocity integration.

Figure 2 illustrates the strike segmentation and subsequent velocity calculation method on a complete data set, i.e., five punches (depicted in Figure 2a). The acceleration along the x, y, and z axes, respectively, is coded on an red-green-blue (RGB) color pattern. Absolute acceleration is indicated by a solid black line. Starting from the identified peak acceleration (Figure 2b), the data was traversed backwards (i.e., in the direction of descending timestamps), until the first acceleration $A_{initial}$ below a user-specified threshold was identified. Visual inspection of the collected acceleration data and the recorded noise resulted in a threshold of 2 m/$s^{2}$. The data segment between $A_{initial}$ and $A_{peak}$ (shown in Figure 2c) represents the strike. In order to obtain velocities, acceleration data was numerically integrated in the form of the trapezoidal rule. This method is a sufficient approximation and requires a relatively small computational effort [16]. The corresponding formula is presented in Equation (1), where *N* is the total number of discrete samples, $x_{i}$ denotes the sample at time *i*, f(x) is the acceleration at time *x*, and Δx is the time interval between successive samples. The result of this velocity integration is presented in Figure 2d.

(1) $$\int_{a}^{b}f\left(x\right)dx\approx \sum_{k=1}^{N}\frac{f\left(x_{k-1}\right)+f\left(x_{k}\right)}{2}\Delta x_{k}$$

For data analysis, both the peak velocity and average velocity were calculated. The peak velocity is the highest impact velocity attained during a valid strike. Conversely, the average velocity is the average across all valid trials.

#### 2.2.1. Measuring Jabs

**Figure 3:**
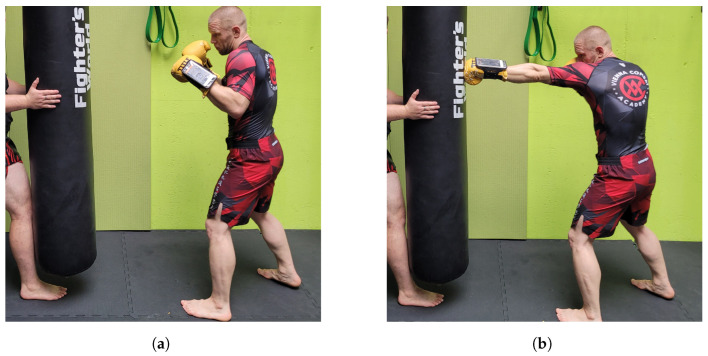
Data collection during the jab punch. (**a**) Guard position; smartphone with IMU attached to the distal wrist; (**b**) execution phase.

For reasons of test standardization, footwork, i.e., stepping forward with the lead foot, was forbidden during punch execution. Furthermore, trunk rotation was discouraged, so that the primary driver of the punch was the extension of the upper limb. During the measurement of jab velocity, hands were covered by 10 Oz amateur competition gloves (Title Boxing LLC, Lenexa, KS, USA). The mobile phone was fixed to the distal wrist of the punching arm and secured in position over the boxing glove strap. In order to minimize movement of the heavy bag and, hence, facilitate the strike recognition process, the bag was steadied by a spotter. Figure 3 illustrates the guard position and the punch execution with the IMU attached to the glove.

#### 2.2.2. Measuring Kicks

For kick velocity testing, shins were covered by standard shin guards, as used in amateur K-1 kickboxing competitions. The mobile phone was fixed to the distal calf of the kicking leg and secured in position over the strap of the shin guard.

**Figure 4:**
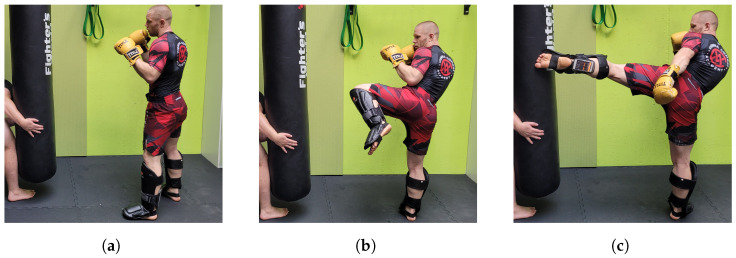
Data collection during the lead roundhouse kick. (**a**) Guard position; smartphone with IMU attached to the distal calf; (**b**) chamber phase; (**c**) extension phase.

Performance of a switch step was forbidden for reasons of test standardization. As in the case of the jab punch, the heavy bag was held in place by a partner in order to minimize bag movement upon impact. Figure 4 illustrates the testing process.

### 2.3. Statistical Analysis

All statistical analyses were carried out using the Jeffreys’s Amazing Statistics Program (JASP) (Version 0.17.2) [31] software. Within-day and between-day reliability and agreement between the two testing sessions were assessed. The within-day coefficient of variation (CV) for each subject was calculated from all valid attempts as $CV\%=\frac{SD}{M}*100$, where SD is the standard deviation between the individual strikes and M is the mean strike velocity.

The between-day CV for each subject was then calculated from the respective (peak or mean) velocities recorded during the familiarization and testing session. In order to detect a systematic bias between the tests, a paired sample *t*-test was performed for all observed striking velocity measures. From the Bland–Altman plots, the 95% LOA between the two and test sessions was calculated for all velocity measures. Additionally, the SEM was calculated based on the mean square from a repeated measures analysis of variance (ANOVA).

To assess relative reliability [32], the two-way mixed, single measure ICC3,1 was calculated, as described by Shrout and Fleiss [33].

## 3. Results

The velocities measured during the trials are presented in Table 2. As described in Section 2.2, peak velocities represent the highest impact velocity attained during a valid strike in a test series. Conversely, the average velocity is the average across all valid trials of the series.

Minimal, mean, and maximal within-day CVs for both sessions are presented in Table 3.

Mean, minimal and maximal between-day CVs for the respective velocity measurement (mean or peak) are reported in Table 4.

**Figure 5:**
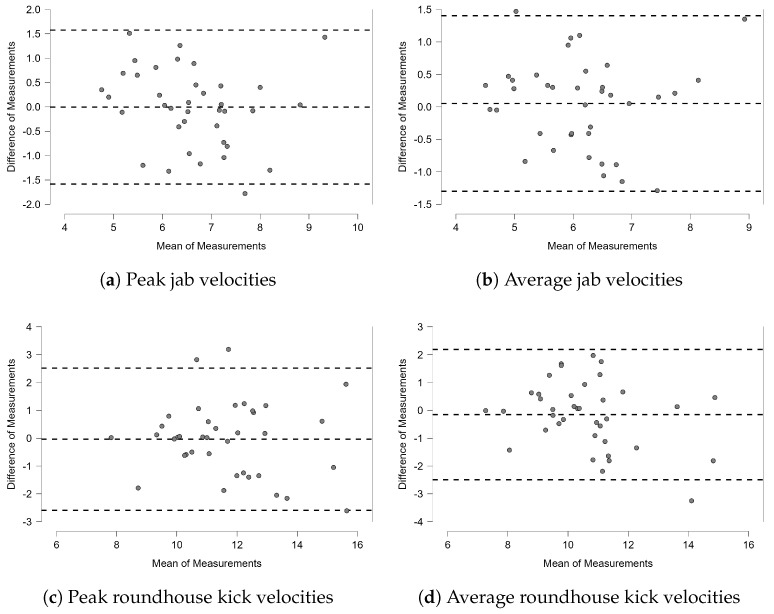
Bland–Altman plot for punch and kick velocities (in m/s). Dashed lines indicate the 95% LOAs, which were set to ±1.96 standard deviations, following the guidelines by Atkinson and Nevill [32].

A paired sample *t*-test revealed no significant differences between the tests for peak (MD = −0.004 m/s, t(37) = −0.032, *p* = 0.974) or average punch velocity (MD = 0.052 m/s, t(37) = 0.464, *p* = 0.646), or for peak (MD = 0.036 m/s, t(37) = −0.172, *p* = 0.864) or average (MD = −0.153 m/s, t(37) = −0.789, *p* = 0.194) kick velocity. The absence of a bias is also confirmed by visual inspection of the corresponding Bland–Altman plots in Figure 5. Mean differences between measurements and the 95% limits of agreement derived from the Bland–Altman plot are summarized in Table 5.

The SEM was found to be between 0.488 and 0.921 m/s, which corresponds to an error of approximately 8 to 9% relative to the population mean. The ICC was found to be between 0.746 and 0.786, depending on the investigated metric. The SEM, the ICC, and the 95% CI for each velocity are outlined in Table 6.

## 4. Discussion

This study was designed to evaluate the feasibility of using a single, low-cost inertial measurement unit (IMU) within a smartphone for capturing movement speed. This system is not intended to serve as a new gold standard but rather as a practical option that provides value to practitioners within its constraints, while this IMU shows promise for tracking velocity trends affordably, further studies are needed to validate its precision in capturing high-speed movements like strikes.

One way of analyzing human movement by way of IMUs is the implementation of a sensor fusion algorithm, which combines the input from inertial sensors with a gyroscope and occasionally a magnetometer [11,21] in order to estimate the pose of the sensor [22].

While sensor fusion and pose estimation are optimal solutions for tracking velocity with high precision, an alternative method using absolute acceleration values has previously been proposed in the literature. Kimm and Thiel [16] implemented this approach, integrating absolute values of acceleration for measuring strike velocity and defining the start and end points of strikes using synchronized video frames. Socci et al. [23] used a similar method in the context of Muay Thai round kicks, by identifying the point of impact as the instant when the absolute acceleration reached its maximum. Unlike Kimm and Thiel [16], Socci et al. [23] did not integrate for velocity, but they used the peak acceleration to mark the moment of impact and subsequently segment the movement in order to evaluate the kinematics of the hip, knee, and ankle. The approach adopted in this study combines the methods proposed by Socci et al. [23], who identified the point of impact, and Kimm and Thiel [16], who integrated the acceleration data to estimate velocity.

The 50 Hz sampling rate was chosen based on hardware restrictions and prior recommendations as a practical threshold for movement analysis, despite not fully meeting the Nyquist–Shannon criterion.

The reliability of the implemented accelerometer-based striking velocity measurement system (ICC3,1 = 0.746 to 0.786, SEM = 0.488 to 0.57 m/s) was shown to be moderate and comparable to commercially available systems [5] when measuring jab velocity.

Harris et al. [5] evaluated the reliability of two commercially available systems (GymAware, Kinetic Performance Technology, Canberra, Australia; and the PUSH Band 2.0, Push Inc., Toronto, ON, Canada) for punch tracking. The GymAware system demonstrated good reliability, with an ICC of 0.871 for mean peak velocity and 0.853 for maximum peak velocity, accompanied by a low coefficient of variation (CV) of 4.21% and standard error of measurement (SEM) values of 0.10 m/s for mean peak velocity and 0.12 m/s for maximum peak velocity. In contrast, the PUSH Band 2.0 exhibited lower reliability, with ICCs of 0.309 for mean peak velocity and 0.227 for maximum peak velocity, a higher CV of 7.44%, and SEM values of 0.24 m/s for mean peak velocity and 0.32 m/s for maximum peak velocity.

In the present study, the SEM ranged from 0.488 to 0.921 m/s, corresponding to an error of approximately 8% to 9% relative to the population mean. The ICC values were found to be between 0.746 and 0.786 depending on the investigated metric. These results are outlined in Table 6. Mean CVs were measured at 5.79% to 8.21% across different conditions, aligning closely with the values reported by Harris et al. [5]. This suggests that the presented system’s reliability is comparable to that of the GymAware for detecting punch performance variability, despite not having the high-end specifications of a commercial IMU. Furthermore, to the best of our knowledge, there are no existing studies investigating the reliability of measuring kick velocities using an IMU, highlighting a gap in the literature that this research aims to address.

Although reliability metrics indicate a promising level of accuracy, it is crucial to consider various confounding factors that could influence these results. In addition to the hardware and software involved in the measurement process, biological and technical variation, as well as issues with test standardization, can contribute to an diminished agreement between measurements. These factors should be considered when evaluating the reliability of the measurement system.

**Biological variation**: Biological variation is usually the main source of within-subject variation [34]. Any change in mental and physical state, such as fatigue, anxiety, or nutrition and hydration status, can potentially influence results in a repeated measurement.
**Hardware**: Noise levels can vary greatly between different specific sensors, even at rest [22]. Different smartphone accelerometers exhibit different levels of validity when compared to gold-standard measurement systems [24]. Studies investigating the LIS3DH sensor used in this study in the context of resistance training have been discussed in Section 2.2.Although movement velocities in punches and kicks are arguably higher than the velocity typically reached in resistance exercise, it seems unlikely that the hardware used in the current study introduces a large potential for measurement error. However, it needs to be acknowledged that a relatively low sampling frequency of 50 Hz was used for data collection.
**Software**: The PhyPhox app, (version 1.1.11) which was used for the measurement of acceleration, has already been shown to be reliable for the measurement of kinematic variables in previous research [28]. Post-processing (i.e., strike segmentation and numerical integration of acceleration data to yield velocity) was performed using a custom algorithm. Numerical integration methods are prone to noise [35], which can potentially arise from issues with test standardization. This inherent sensitivity to noise is a recognized limitation of the current approach.
**Standardization**: The sensitivity of the algorithm to noise is closely tied to the technical execution of the investigated striking techniques and the standardization of the protocol. Steps have been undertaken to eliminate as much variation from the test protocol as possible. The use of footwork or trunk rotation while delivering the striking techniques was explicitly forbidden. However, these constraints are in conflict with the way full contact fighters usually perform their technique. Slight counter movements, trunk rotations, or weight shifts which are not detectable under loose clothing can introduce noise to the acceleration measurement and, hence, compromise agreement between repeated measures. Technological issues such as noise notwithstanding, a slightly altered technique due to insufficient test standardization may result in a changed velocity.
**Technical variation**: Human movement is inherently variable. Cowin et al. [36] (p. 2) pointed out that ”even highly skilled individuals who consistently achieve the same outcome show movement variability within a goal-oriented action or task”Punching technique has been shown to be subject to outcome variability, even in highly trained individuals. Dunn et al. [37] investigated the accuracy and reliability of a punch measurement system and a boxing-specific punch test with a population of amateur boxers who were ranked first or second in Australia in their respective division. A high outcome variability (CV = 4.4% to 13.6%) was observed for kinetic variables associated with jab impact force. In contrast, the measurement system was shown to demonstrate excellent reliability (ICC = 0.999, 95% CI [0.999, 0.999], *p* < 0.001) and agreement (typical error = 0.8 N, CV = 0.05%). The inherent variability of the task being measured imposes an implicit natural upper limit on the reliability of the associated measurement system.

The described sources of biological and technical variability are inherent to human movement and present a challenge for all diagnostic tools, including gold-standard systems. Although efforts were made to minimize variability through protocol standardization, complete elimination is not feasible in practice, as movement variability is a fundamental characteristic of human motion. This limitation underscores the complexity faced by any motion analysis method in capturing highly dynamic and variable actions accurately.

The SEM observed in the repeated measurements (0.488 m/s to 0.844 m/s) was found to be low (8 to 9%) with respect to the respective SD (1.058 m/s to 2.005 m/s). This suggests a low random error. Conversely, within-day CV values for velocity measurements ranged from 0.67% to 17.55%, which is comparable to the results obtained by Dunn et al. [37], while between-day CV values ranged from 0.15% to 20.73%

A combination of relatively low SEM and high CV values can be observed when the measurements are precise on average, but there are certain cases or subgroups within the data where the variability is significantly higher. As with the power measures, this inconsistency can make it challenging to draw meaningful conclusions from the correlation analysis.

Measurements of average jab and kick velocities in this study demonstrated slightly higher reliability and agreement than measurements of peak jab and kick velocities. This can in part be explained by the fact that averaging all valid attempts smooths out noise stemming from the above mentioned factors [38]. The comparison between peak and average velocity scores, as shown in Table 2, demonstrates the potential discrepancy between a single maximum value and the average performance. Single maximum values may represent an athlete’s best effort but could also reflect random fluctuations that are not consistently reproducible across trials. This variability poses a limitation in using peak values alone for tracking performance trends. Consequently, average values provide a more stable indicator of performance, though they may underrepresent an athlete’s true peak capacity.

Given the high deviations observed in striking velocity measurements, it is recommended to use average values from a series of strikes to track changes in performance over time. Averaging can effectively smooth out random fluctuations, providing a clearer trend of performance changes rather than focusing on isolated data points that may be affected by variability. However, it is important to recognize that, while this approach enhances the reliability of detecting trends, it may not always align with the objectives of diagnostic measures, particularly in contexts where capturing individual variability or peak performance is critical. Therefore, while averages offer a valuable tool for monitoring long-term progress, their use should be balanced with an understanding of the potential trade-offs in accurately reflecting real-time performance. Furthermore, care must be taken to manage fatigue when using a multiple-repetition testing protocol to measure power-dominant activities such as punches or kicks [38] (p. 227).

A major limitation of this study is the absence of validation against a widely recognized method, such as three-dimensional (3D)motion capture, to establish measurement accuracy. However, the recent literature underscores the current lack of a universally accepted “gold standard” for punch performance assessment in combat sports. Lenetsky et al. [9] suggested that, in the absence of such a standard, researchers should choose methods based on practical considerations, including economic and organizational needs, while adhering to measurement principles. Given this context, the present study focuses on evaluating the feasibility and consistency of a low-cost IMU-based device as an accessible solution for strike performance measurement. Future research will aim to further validate this system by comparing it with established methods, supporting its potential to reliably capture punch performance in practical settings.

A further limitation of the current study is that the velocity measurements are calculated in the local IMU reference system, rather than a global reference frame; while this may not fully represent the velocity in a global context, the linear nature of the movements analyzed (e.g., punches and kicks) suggests that this limitation is unlikely to have a substantial effect on the velocity estimates.

Another factor contributing to uncertainty regarding the validity of the measurement system stems from the low sampling frequency. Minakov and Passerone [13] reported an impact duration of 15 ms when experienced amateur boxers threw straight punches at a heavy bag. This means that, in order for the strike to be detected in an IMU measurement, a sampling frequency of at least 67 Hz is necessary [15]. In fact, the Nyquist–Shannon sampling theorem states that, in order to completely reconstruct a signal where the highest appearing frequency is B, a sampling rate (Nyquist rate) of at least 2B is necessary [39], which amounts to 134 Hz in the above example. The fact that, due to technical constraints, the sampling frequency in this study was only 50 Hz, poses a considerable limitation. This limitation can in practice be observed in commercially available solutions, too. In one study [5], the GymAware was used to sample punch acceleration at 200 Hz. Before analysis, however, the signal was down-sampled to 50 Hz. The authors of the study concluded that it is uncertain whether the sampling frequency is adequate for accurately measuring punch velocity. However, they also pointed to force-plate based research [40] in the field of athletic movements, where frequencies of 25 to 100 Hz are deemed adequate for analysis. Future research is needed to determine the lowest and optimal (from a cost-benefit consideration) sampling frequency.

## 5. Conclusions

In conclusion, low-cost IMU based velocity measurement devices can be employed to track changes in punch and kick velocity in martial artists. However, due to the technological limitations and inherent biological variation in complex sports movements, practitioners should consider using average results over a series of strikes rather than relying on a single maximum value, which may be skewed. While averaging can enhance the reliability of measurements by reducing random fluctuations, it typically underestimates the true peak performance, potentially compromising validity. Therefore, practitioners should be aware of this trade-off and apply averages judiciously, particularly in contexts where capturing maximum performance is critical. Additionally, more research on the concurrent validity of these measurements, especially in comparison with established technologies like motion tracking, is necessary.

## Figures and Tables

**Table 1:** Characteristics of the CS athletes that completed the study; Y: years, M: male, F: female.

	Age (Y)		Body Mass (kg)		Height (cm)		Experience (Y)	
	**F**	**M**	**F**	**M**	**F**	**M**	**F**	**M**
Mean	32.17	32.85	64.42	81.73	166.54	177.77	7.33	9.23
Std. Deviation	8.23	9.43	6.83	14.39	6.87	6.95	4.31	6.96
Minimum	21.00	18.00	51.00	60.00	158.50	167.00	2.00	1.00
Maximum	44.00	56.00	75.00	118.00	178.00	193.00	18.00	30.00

**Table 2:** Punch and kick velocities (m/s); Jab Out: punching velocity, Kick Out: kicking velocity, avg: average.

	Jab Out Avg	Jab Out Peak	Kick Out Avg	Kick Out Peak
Mean	6.11	6.63	10.73	11.67
Std. Deviation	1.06	1.19	2.01	2.01
Minimum	4.29	4.57	7.27	7.81
Maximum	8.25	8.85	16.00	16.95

**Table 3:** Within-day strike velocity coefficient of variation; Jab Out: punching velocity (in m/s), Kick Out: kicking velocity (in m/s).

	Jab Out Session 1	Kick Out Session 1	Jab Out Session 2	Kick Out Session 2
Mean	5.79%	8.21%	6.69%	7.46%
Min	2.13%	0.67%	1.73%	2.32%
Max	12.79%	17.81%	14.11%	17.55%

**Table 4:** Between-day strike velocity coefficient of variation; Jab Out: punching velocity, Kick Out: kicking velocity, avg: average.

	Jab Out Avg	Kick Out Avg	Jab Out Peak	Kick Out Peak
Mean	6.43%	5.96%	6.74%	5.75%
Min	0.34%	0.15%	0.32%	0.13%
Max	20.73%	16.27%	20.05%	19.25%

**Table 5:** 95% limits of agreement for strike velocities in m/s; Jab Out: punching velocity (in m/s), Kick Out: kicking velocity (in m/s), SD: standard deviation.

	Mean Difference	Mean Diff. + 1.96 SD	Mean Diff. − 1.96 SD
Jab Out Avg	0.052	1.403	−1.299
Kick Out Avg	−0.153	2.187	−2.493
Jab Out Peak	−0.004	1.576	−1.584
Kick Out Peak	−0.036	2.517	−2.590

**Table 6:** Standard error of measurement (SEM) and intraclass correlation coefficient (ICC) for striking velocity measurement; Jab Out: punching velocity, Kick Out: kicking velocity, CI: confidence interval, avg: average.

	SEM m/s	SEM%	ICC3, 1	95% CI
Jab Out Avg	0.488	∼8	0.786	[0.625, 0.883]
Kick Out Avg	0.844	∼8	0.784	[0.622, 0.881]
Jab Out Peak	0.570	∼9	0.746	[0.563, 0.860]
Kick Out Peak	0.921	∼8	0.778	[0.613, 0.878]

## Data Availability

The original contributions presented in the study are included in the article; further inquiries can be directed to the corresponding author.

## Abbreviations

The following abbreviations are used in this manuscript:

ANOVA	Analysis of Variance
CI	Confidence Interval
CS	Combat sports
CV	Coefficient of variation
ICC	Intraclass correlation coefficient
IMU	Inertial measurement unit
JASP	Jeffreys’s Amazing Statistics Program
LOA	Limits of agreement
MD	Mean difference
MMA	Mixed Martial Arts
SEM	Standard error of measurement
